# Flexible Textile Sensors-Based Smart T-Shirt for Respiratory Monitoring: Design, Development, and Preliminary Validation

**DOI:** 10.3390/s24062018

**Published:** 2024-03-21

**Authors:** Chiara Romano, Daniela Lo Presti, Sergio Silvestri, Emiliano Schena, Carlo Massaroni

**Affiliations:** 1 Research Unit of Measurements and Biomedical Instrumentation, Departmental of Engineering, Università Campus Bio-Medico di Roma, Via Alvaro del Portillo, 21, 00128 Rome, Italy; c.romano@unicampus.it (C.R.); d.lopresti@unicampus.it (D.L.P.); s.silvestri@unicampus.it (S.S.); e.schena@unicampus.it (E.S.); 2 Fondazione Policlinico Universitario Campus Bio-Medico, Via Alvaro del Portillo, 200, 00128 Rome, Italy

**Keywords:** wearable device, conductive textiles, flexible sensors, breathing monitoring

## Abstract

Respiratory rate ($f_{R}$) monitoring through wearable devices is crucial in several scenarios, providing insights into well-being and sports performance while minimizing interference with daily activities. Strain sensors embedded into garments stand out but require thorough investigation for optimal deployment. Optimal sensor positioning is often overlooked, and when addressed, the quality of the respiratory signal is neglected. Additionally, sensor metrological characterization after sensor integration is often omitted. In this study, we present the design, development, and feasibility assessment of a smart t-shirt embedded with two flexible sensors for $f_{R}$ monitoring. Guided by a motion capture system, optimal sensor design and position on the chest wall were defined, considering both signal magnitude and quality. The sensors were developed, embedded into the wearable system, and metrologically characterized, demonstrating a remarkable response to both static (sensitivity 9.4$\;\Omega \cdot {\%}^{-1}$ and 9.1$\;\Omega \cdot {\%}^{-1}\;$for sensor A and sensor B, respectively) and cyclic loads (min. hysteresis span 20.4% at 36 bpm obtained for sensor A). The feasibility of the wearable system was assessed on healthy volunteers both under static and dynamic conditions (such as running, walking, and climbing stairs). A mean absolute error of 0.32 bpm was obtained by averaging all subjects and tests using the combination of the two sensors. This value was lower than that obtained using both sensor A (0.53 bpm) and sensor B (0.78 bpm) individually. Our study highlights the importance of signal amplitude and quality in optimal sensor placement evaluation, as well as the characterization of the embedded sensors for metrological assessment.

## 1. Introduction

In the realm of healthcare and sports science, the monitoring of breathing patterns has garnered increasing attention due to its implications for overall well-being and athletic performance [1]. In fact, continuous monitoring of breathing patterns can offer invaluable information on medical conditions, ranging from respiratory disorders to cardiovascular issues [2,3]. Moreover, respiratory rate ($f_{R}$) is intrinsically connected to stress and mental well-being; therefore, monitoring $f_{R}\;$can aid in stress management and the assessment of mental health conditions [4,5]. In the realm of sports, continuous $f_{R}$ monitoring takes on a pivotal role in sustaining physical exertion. Monitoring the breathing patterns of athletes provides insight for the assessment of endurance levels and the customization of training programs. Additionally, it plays a key role in optimizing training loads, determining recovery periods, and fine-tuning athletic performance [6].

In this context, the introduction of wearable devices for continuous monitoring of $f_{R}\;$represents a significant step forward, thanks to several advantages over traditional technologies [7]. The crucial aspect lies in their ability to provide real-time feedback, enabling adjustments for stress management, physical activity adaptation, and general health monitoring. The portability and unobtrusiveness exhibited by these devices contribute to their widespread use, allowing individuals to wear them during activities of daily living [8]. In the sports context, where comfort and performance optimization are paramount, wearable sensors for continuous $f_{R}\;$monitoring play a pivotal role. For example, coaches and athletes can receive instantaneous feedback on $f_{R}$, allowing for adjustments in training loads and recovery periods.

Hence, the benefits due to the widespread adoption of wearable devices for $f_{R}\;$monitoring in sports has led to the development of different types of devices integrating various sensor technologies [7]. For instance, face-mounted sensors, such as temperature sensors, pressure sensors, or flow sensors, designed to capture exhaled airflow, are finding great acceptance [9,10]. Moreover, a widely embraced class of wearable devices for respiratory monitoring in sports includes chest-worn devices, as they allow minimizing the impact on the athlete’s movements. These solutions may embed sensors such as accelerometers [11], piezoresistive textiles [12,13,14], capacitive sensors [15,16], and ionic gen sensors [17] to detect chest movements associated with respiratory activity. Among all, textile sensors stand out because they can be manufactured in different shapes and easily integrated into garments. In addition, these sensors require simple interface electronics, helping to reduce the overall footprint of the system. This feature increases their suitability in sports, ensuring minimal disruption of the athlete’s movements while maintaining efficient $f_{R}\;$monitoring capabilities. Various wearable devices leveraging this technology have been suggested in the literature. For instance, piezoresistive textile sensors embedded into headbands or t-shirts and placed at different locations on the rib cage have been proposed [18]. However, although the importance of sensor placement on the rib cage for respiratory monitoring has been highlighted [18,19], the identification of optimal sensor placement to optimize performance in $f_{R}$ monitoring using this technology is typically neglected or based only on the amplitude of chest deformations during breathing [20]. However, it is crucial to recognize that greater signal amplitude does not always result in greater signal reliability. The quality of the respiratory signal collected in different areas of the chest is also relevant, as it indicates how much each signal is affected by artefacts.

Our study had a threefold objective. First, guided by an optoelectronic system with passive retro-reflective markers, we proposed an investigation of the deformations exhibited by the chest wall during breathing. Both the amplitude and the quality of the respiratory signal collected in the different areas of the chest were evaluated in order to identify two optimal measurement sites for respiratory monitoring. The information on signal amplitude guided us in the selection of the sensor design and chest wall locations most subject to deformation during breathing. Assessing signal quality allowed us to exclude areas that were most affected by artefacts. This analysis resulted in the identification of optimal measurement sites for $f_{R}\;$monitoring with a strain sensor. In addition, it provided guidance on the dimensions of the sensors to be developed as well as the parameters to be set for the metrological characterization of these elements. Secondly, based on the results of the first investigation, we designed and developed two piezoresistive textile sensors. Subsequently, they were embedded in a wearable t-shirt via a polymer matrix and metrologically characterized. This last step, performed after the integration of the sensors, was undertaken to enhance the reliability of assessing the calibration curve and dynamic behavior of the sensors. In fact, many studies merely characterize the sensor before integration into the wearable device, neglecting potential changes in metrological characteristics after integration [18]. Finally, a pilot study was carried out to assess the feasibility of the proposed system in monitoring $f_{R}$ during both static and dynamic tests.

## 2. Chest Wall Deformation Analysis for Optimal Sensor Placement

Piezoresistive sensors operate on the principle of the piezoresistive effect, according to which changes in the electrical resistance of semiconductor materials, such as silicon, occur in response to mechanical deformation [21].

In the context of respiratory rate monitoring, piezoresistive sensors are placed on the chest to detect its deformation during inhalation and exhalation. As the chest expands and contracts, the attached sensors undergo mechanical strain that leads to changes in electrical resistance [7]. Due to their working principle, the optimized placement of piezoresistive sensors on the chest assumes paramount importance, as preliminary investigated in [22].

To identify the optimal position for capturing the respiratory waveform using piezoresistive textile sensors, an analysis of the deformations exhibited by the chest wall during breathing was carried out.

### 2.1. Experimental Setup and Protocol

**Figure 1:**
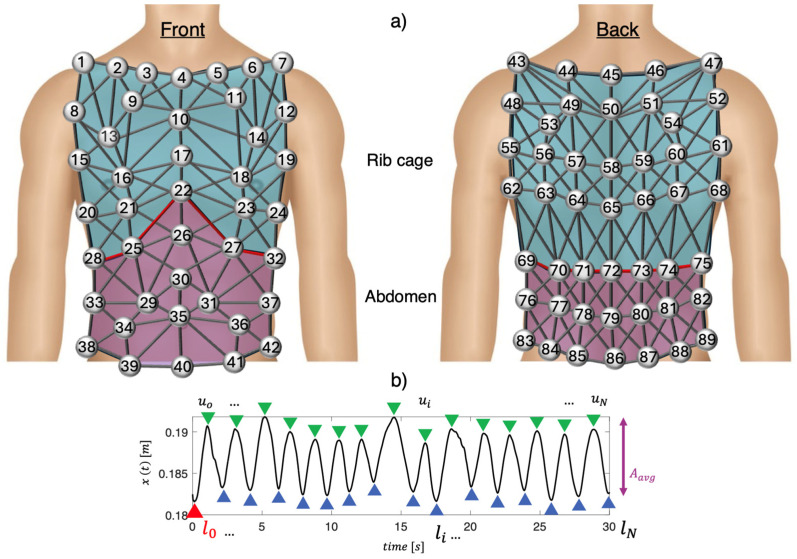
(**a**) Illustration depicting the spatial distribution of 89 photo-reflective markers on the chest wall, segmented into the abdomen (depicted in violet) and rib cage (depicted in blue). The diagram also outlines the connections between pairs of markers; (**b**) Representation of a relative displacement signal (i.e., x) between two markers over time, with the identification of maximum (end of inhalation, depicted as green triangles) and minimum (end of exhalation, depicted as blue triangles) peaks.

Experimental trials involved five male trained volunteers (mean age: 31 ± 4 years, body mass: 65 ± 8 kg, height: 173 ± 4 cm). The setup included a motion capture system (Qualisys AB, Sweden) with 10 infrared digital cameras arranged in a 360° circular pattern about 3 m from each participant. Eighty-nine photo-reflective hemispherical markers were placed on the chest wall and back, following a specific protocol, as described in [22,23]. In Figure 1a is shown a schematic representation of the marker positioning.

Participants were asked to breathe quietly while seated on an upright cycle ergometer (Lode Corival, Groningen, The Netherlands). Chest wall movements were continuously recorded over a 30 s duration.

The study received approval from the University of Kent, School of Sport and Exercise Sciences’ Local Research Ethics Committee in Chatham Maritime, UK (Reference Number: Prop17_2013_14). The study adhered to the principles of the Declaration of Helsinki at all stages, and each participant provided written informed consent to participate.

### 2.2. Data Analysis and Results

Following data collection, a previously described geometric model [22] was exploited to define 82 prisms and 279 links between the 89 markers attached to the subjects’ chests, as shown in Figure 1a. The chest wall was divided into two compartments: the abdomen (violet in Figure 1a) and the rib cage (blue in Figure 1a).

Before delving into the process of selecting the optimal positions for respiratory monitoring using piezoresistive textile sensors, it is essential to clarify the operating principle of these sensors in respiratory monitoring. The optimal sensor positions were investigated by analyzing each link between two markers, considering two main aspects: (1) the positions on the rib cage that exhibited greater deformations during respiration were chosen as the most reliable for respiratory monitoring with piezoresistive sensors. In fact, the greater the deformation of the rib cage during respiration, the wider was the change in resistance of the piezoresistive textile sensors; (2) the positions on the rib cage with higher signal-to-noise ratios (SNRs) were considered as the most reliable for respiratory monitoring. Subsequently, to identify the links with the maximum deformations for each subject, the following steps were performed for both the rib cage and abdomen:

(i) The Euclidean distance of each pair of markers over time was calculated to assess the relative displacement of each link (i.e., x(t)). The signal $x\left(t\right)$ was then filtered using a 1st order Butterworth bandpass filter with cut-off frequencies of 0.01 Hz and 2 Hz to remove frequencies unrelated to respiratory activity. An example of the filtered x(t) is shown in Figure 1b;
(ii) The average amplitude (i.e., $A_{avg}$) of $x\left(t\right)$ was calculated by computing the mean difference between each maximum peak (i.e., $x\left(u_{i}\right)$ at the end of inhalation) and the next minimum peak (i.e., $x\left(l_{i}\right)$ at the end of exhalation). An example is shown in Figure 1b;
(iii) The ratio between $A_{avg}$ and $x\left(t\right)$ calculated at the end of the first inhalation ($l_{0}$) was computed to obtain the strain value (i.e., ε), as follows:
  (1) $$\varepsilon \%=\frac{A_{avg}}{l_{0}}\cdot 100$$

Then, the ε values were normalized for each subject and subsequently averaged (i.e., $\varepsilon_{avg}^{norm}$). This process was carried out separately for the abdomen and rib cage. Hence, per each compartment, we assigned to the links values ranging from 100% (related to the link with the maximum $\varepsilon_{avg}^{norm}$) to 0% (related to the link with the minimum $\varepsilon_{avg}^{norm}$). To verify that $\varepsilon_{avg}^{norm}$ was attributable to respiratory activity, the signal-to-noise ratio of x(t) was evaluated for each link. Hence, for each subject, the rib cage volume signal as described in [22] was used to define the subject’s $f_{R}$ mean value during the whole trial. The power spectral density (PSD) was calculated and the frequency corresponding to the maximum PSD was selected as the mean $f_{R}$. Then, frequencies between $f_{R}^{mean}\pm \;10\%$ were considered as *signal*, while all frequencies outside this band were considered as *noise*. Accordingly,

(i) The PSD value of the signal x(t) for each link was calculated;
(ii) The average value of the PSD in the range of frequencies identifying the signal and the average value of the PSD within the frequency range identifying the noise were calculated;
(iii) The SNR ratio for each link was calculated. Thereafter, SNR values were normalized for each subject and each compartment. Then, they were added to the $\varepsilon_{avg}^{norm}$ values found from the previous analysis and normalized to obtain $U_{avg}^{norm}$.

**Figure 2:**
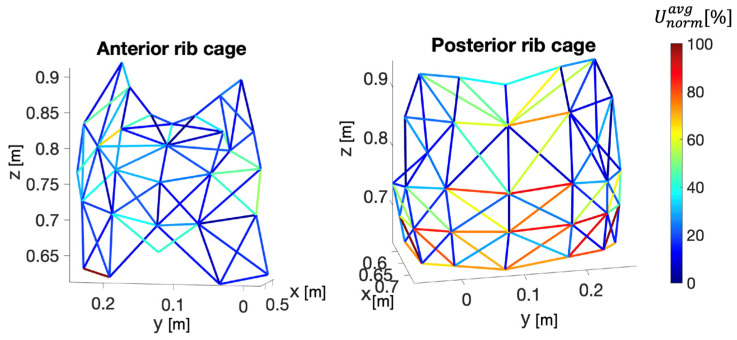
Representation of the connections among markers placed on both the anterior and posterior rib cage. The color map corresponds to $U_{avg}^{norm}$ values, representing the combination of deformation and normalized SNR values.

Overall, the obtained results suggested that the most reliable locations for placing piezoresistive sensors for respiratory monitoring corresponded to the links connecting markers m25–m28 ($\varepsilon_{avg}\%:\;5.0\pm 2.3$, $l_{0}:83.1\;mm$) and markers m66–m67 ($\varepsilon_{avg}\%:0.68\pm 0.22,\;l_{0}:73.3\;mm$), as shown in Figure 2. Two positions for the sensors were chosen on the same side of the chest. This configuration allowed one side of the t-shirt to remain sensor-free, facilitating the implementation of a size adjustment system to suit the different anthropometries of the subjects. Our investigation’s findings enabled us to identify the sensors’ length, location within the wearable device, and the deformation they undergo during breathing. This information was useful for the sensors’ metrological characterization.

## 3. Development of Wearable System and Metrological Characterization

### 3.1. Sensor Development and Integration into the Wearable System

Based on the results obtained from the previous analysis, the design of the two sensing elements was carried out. For the development of the sensors, we employed a conductive piezoresistive textile (MedTex P130) characterized by a sheet resistance of 0.15 Ω/square. Hence, placed on the subject’s chest wall, these sensors could be used to collect the breathing waveform based on the movements exhibited by the chest wall during breathing. At the end of exhalation, the volume of the chest wall was denoted as $V_{0}$ and the sensors’ resistance as $R_{0}$. During inhalation, the volume of the rib cage increased until the end of inhalation was reached ($V_{1}$). During this phase, the sensor’s resistance increased until it reached a value of $R_{1}>R_{0}$.

**Figure 3:**
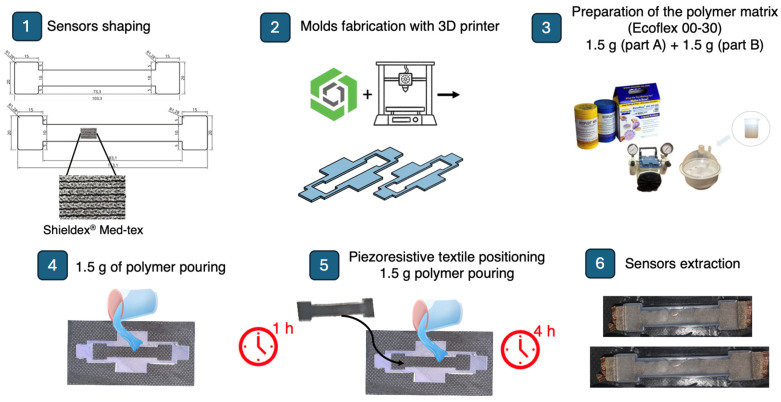
Schematization of the main steps carried out for the realization the two piezoresistive textile sensors and the integration into the wearable system by means of a polymer matrix.

The shape of the sensors was made such that there was a central part 10 mm in height, while the length was made as indicated by the previous analysis (83.1 mm and 73.3 mm for the two sensors, respectively). In addition, two side rectangles (20 mm in height, 10 mm in length) were made to improve the adhesion of the sensor to the overall wearable system. Then, the sensors were embedded into the t-shirt by means of a polymer matrix (Ecoflex 00-30—SmoothOn, Inc., Macungie, PA, USA) with 3 mm thickness to reduce the influence of external factors such as moisture and to improve the robustness. For this purpose, two molds were made of polylactic acid (PLA) by means of a 3D printer (Creality Ender-3 V2). The dimensions of the two molds are shown in Figure 3. The 3D mold was placed on a sports fabric t-shirt at the positions identified by the analysis described in the previous section and attached to it by double-sided tape. Ecoflex 00-30, with a weight ratio 1:1 between part A and part B, was firstly degassed using a vacuum/pressure pump (mod. VCP 130, VWR International, LLC, Radnor, PA, USA) to remove air bubbles. Then, the mixture was cast into the 3D molds up to a height of 1.5 mm, followed by a waiting period of 1 h for curing. Subsequently, the piezoresistive textiles were placed inside the molds and Ecoflex 00-30 (1.5 g) was poured until a total mold height of 3 mm was reached. Finally, a curing period of 3 h was observed to ensure thorough drying of the polymer matrix, in accordance with the manufacturer’s guidelines [24].

The unloading resistance of the sensor at position m25–m28 was 23 Ω, while that of the sensor at position m66–m67 was 22 Ω. The sensors embedded into the wearable system are shown in Figure 3.

### 3.2. Metrological Characterization

After integration, the sensors were metrologically characterized by application of both a static tensile load and cyclic loading and unloading cycles. For both characterizations, a mechanical tensile machine (model 3365, Instron, MA, USA) equipped with a 500 N load cell was employed to exert axial strain on the developed sensors (acquisition frequency: 100 Hz). A voltage divider with a fixed resistance R1 of 30 Ω was used to convert the sensors’ output (resistance variations) into voltage variations (Vo). An external data acquisition board (NI—DAQ 6009, National Instruments, Austin, TX, USA) was used to power the voltage divider and acquire Vo (acquisition frequency: 100 Hz). Two anchoring clamps, made of PLA material using 3D printing technology, were realized to improve the sensors’ grip to the clamps of the testing machine.

The static assessment of the flexible sensors was carried out by performing tensile tests by means of a mechanical tensile machine used to apply ε values (from 0% to 10%) in quasi-static conditions (displacement rate: 5 mm/min). Hence, each flexible sensor provided with the PLA anchor systems was placed between the two clamps of the testing machine, and a total of five tensile tests were carried out, straining the specimen up to 10% of its initial length (83.1 mm for the m25–m28 link—sensor A, and 73.3 mm for the m66–m67 link—sensor B).

**Figure 4:**
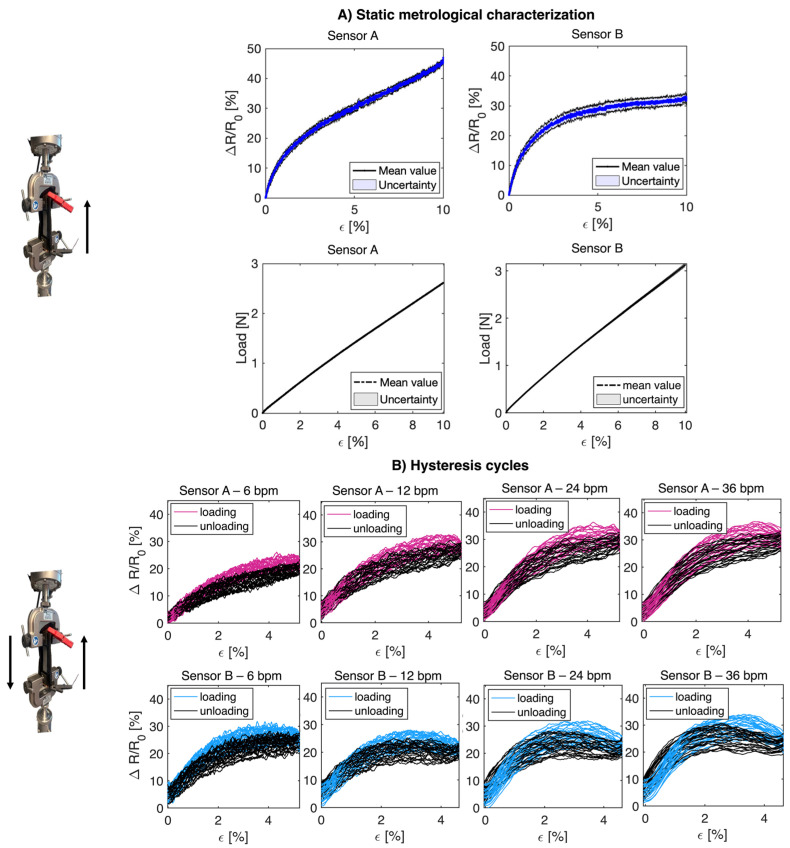
(**A**) The upper panels represent the output of the flexible sensors integrated into the wearable system (sensor A and sensor B) following the application of a quasi-static tensile load up to a maximum strain equal to 10% of the initial length of each sensor; the lower panels represent the plot of the tensile load applied to the sensors as a function of their deformation. (**B**) Representation of the hysteresis cycles obtained by applying cyclic loads to the two flexible sensors simulating different frequencies: 6 bpm, 12 bpm, 24 bpm, and 36 bpm.

Changes in sensor resistance (Δ*R*) were collected during each trial with the acquisition board. The calibration curve (ΔR vs. *ε*) was obtained by processing the collected data through a customized algorithm in MATLAB R2021a, as reported in Figure 4. The mean value of ΔR and relative uncertainty over the five trials were calculated, the latter considering a t-student distribution with four degrees of freedom and a 95% confidence level. The static sensitivity ($s_{\varepsilon }$) of the sensors was calculated considering two different values of $\varepsilon_{max}\;$(5% and 10%, respectively), as in the following equation:

(2) $$S_{\varepsilon }(\varepsilon_{max})=\frac{\Delta R}{\varepsilon_{max}}$$

Moreover, sensitivity to strain was assessed by calculating the sensors’ gauge factor (*GF*) as follows:

(3) $$GF=\frac{\Delta R/R_{0}}{\varepsilon_{max}}$$

In addition, the mechanical properties of the sensors were evaluated in order to understand the load exerted on the sensors. Thus, the tensile load required to induce the analyzed deformation (up to 10%) was evaluated for each sensor. Figure 4A shows the tensile load values as a function of the deformation undergone by the sensor.

Results related to m25–m28 link (sensor A) and m66–m67 link (sensor B) for $S_{\varepsilon }\;$(5%) and $S_{\varepsilon }\;$(10%), GF, and the applied load are shown in Table 1.

The flexible sensors underwent 30 loading and unloading cycles to evaluate their response when subjected to cyclic loads to mimic the movement of the chest wall during breathing. The specimen was placed between the clamps of the tensile machine and was strained and unstrained repeatedly. Concurrently, data were collected at 100 Hz with the data acquisition board. A maximum strain of 5% of the initial length of the sensor was set (4.2 mm for the m25–m28 link and 3.7 mm for the m66–m67 link). The chosen strain value was determined based on the preliminary analysis, revealing that the average chest deformation induced by respiration never exceeded 5% (see Section 2). Also, four different velocities simulating $f_{R}$ values of 6, 12, 24, and 36 breaths per minute (bpm) were used for the loading and unloading cycles to include bradypnea, eupnea, and tachypnea. The hysteresis span was then computed as described below [25]:

(4) $${\%e}_{H}=\frac{e_{max}}{r_{FS}}\cdot 100$$

where $e_{max}\;$denotes the maximum difference between the values of the resistance changes during the loading and unloading cycles at the same ε value, and $r_{FS}$ is the sensor’s full-scale of each loop. Results related to sensor A and sensor B were calculated and are shown in Table 2.

Furthermore, an increase in the resistance change ratio with frequency can be seen from Figure 4B, as highlighted in previous studies [26,27]. Additionally, this phenomenon of increasing resistance change ratio with frequency may also have contributed to the observed decrease in the ${\%e}_{H}$ values with increasing strain rate during dynamic tensile tests, as shown in Table 2.

## 4. Pilot Study on Healthy Volunteers

### 4.1. Experimental Setup and Protocol

A feasibility analysis was carried out through pilot tests on eight healthy volunteers (2 men and 6 women, height 169 ± 7 cm, weight 62 ± 9 kg, expressed as mean ± standard deviation) under different operating conditions, both static (i.e., sitting and standing) and dynamic (i.e., walking, running, and climbing stairs). For this purpose, a signal conditioning board was developed with Autodesk’s EAGLE version 9.6.0 software for resistance–voltage conversion. The latter was then connected with the electronic board Feather M0 Adalogger (Adafruit, New York, NY, USA) used for digitizing and storing data with an acquisition frequency of 25 Hz. The voltage values for each sensor were then saved to microSD and then analyzed in the MATLAB^®^ environment. Both the signal conditioning board and the storage board were fitted in a 3D-printed PLA case. The latter was then placed on the back of the wearable system, and the connection between the conditioning board and the two sensors was made by means of flexible cables.

**Figure 5:**
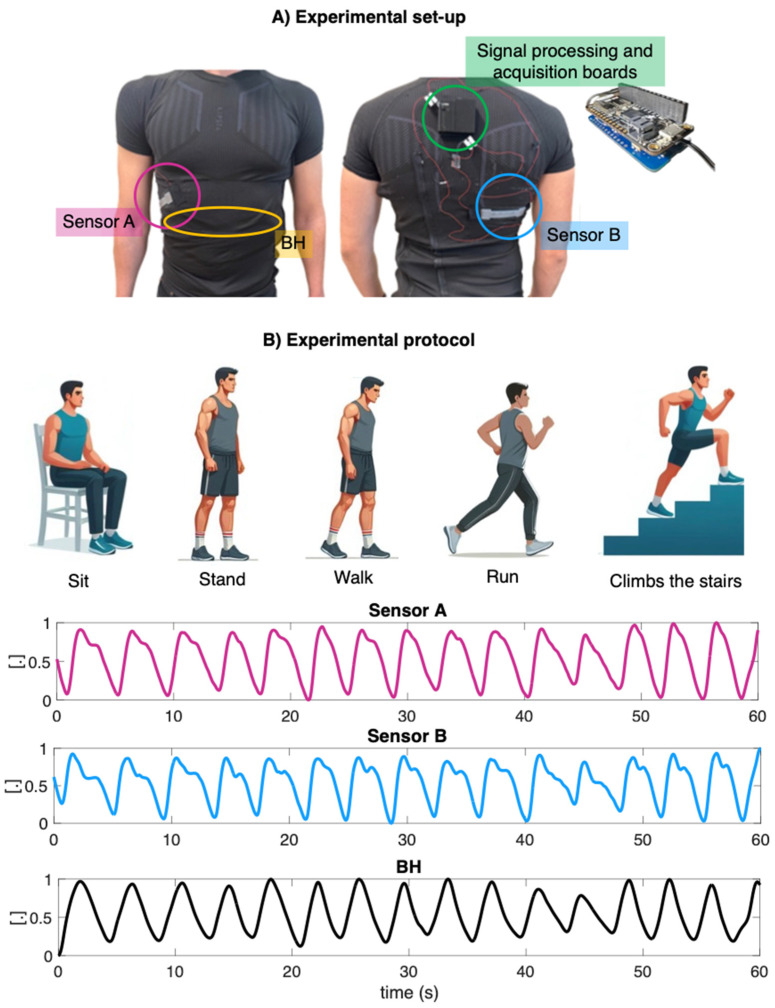
(**A**) Experimental setup consisting of the two flexible sensors embedded into the wearable system and a PLA case placed on the back of the wearable system, which contains both the signal conditioning board for resistance-voltage conversion and the board for data acquisition and storage. Simultaneously, the subject wears the BH system used to collect the reference respiratory wave. (**B**) Experimental protocol consisting of both static and dynamic phases. An example of the signals collected with the two flexible sensors (sensor A in magenta and sensor B in blue) integrated into the wearable system and with the reference system (depicted in black) during the standing phase is given in the panels below.

The experimental setup (Figure 5A) consisted of the whole developed wearable system and a wearable chest strap (Zephyr Bioharness 3.0, by Medtronic, hereinafter BH) to collect the reference respiratory waveform with an acquisition frequency of 25 Hz (accuracy of ±3 bpm for low and moderate activities) [28]. Therefore, each volunteer was asked to wear the wearable system and carry out the following experimental protocol: 10 s of apnea for synchronization of the developed wearable system with the reference system, 60 s of uncontrolled breathing and 60 s of tachypnea in sitting position, 60 s of uncontrolled breathing and 60 s of tachypnea in standing position, and 60 s of walking with uncontrolled breathing. Moreover, we asked the volunteer to perform 50 s of running and 50 s of climbing stairs, as depicted in Figure 5B.

### 4.2. Data Analysis

The collected data were analyzed in the MATLAB^®^ environment. At first, the data gathered from the two flexible sensors and with the reference system were synchronized by cutting them from the endpoint of inhalation following the apnea performed during the first part of the protocol. Subsequently, signal components not related to respiratory activity were attenuated using a first-order Butterworth bandpass filter with cut-off frequencies of 0.01 Hz and 1.5 Hz. Finally, to obtain the mean $f_{R}$ in each trial, for signals collected with the sensors positioned on the m25–m28 link (sensor A) and m66–m67 link (sensor B) and the reference system, the power spectral density (PSD) was estimated with 0.01 bpm of resolution. The frequency corresponding to the maximum peak PSD was selected and multiplied by 60 to obtain the mean $f_{R}$, expressed in bpm. Therefore, we obtained the mean $f_{R}$ values for each subject and each stage of the protocol. In addition, the same $f_{R}$ assessment was performed by considering the signal obtained as the sum of the signals of the two flexible sensors.

Next, the errors of the proposed wearable system compared with the reference system were calculated for each stage of the protocol, averaging across all subjects, and considering both the sensors separately and combining them. Finally, we computed the mean absolute error (MAE) per each phase of the protocol.

### 4.3. Results

**Figure 6:**
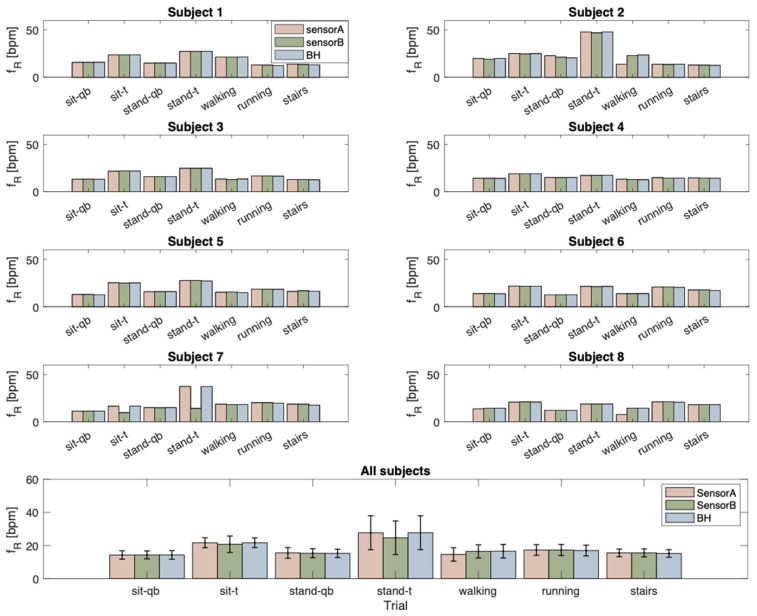
In the upper panels, bar charts are displayed for each subject. The color representation is as follows: orange corresponds to the $f_{R}$ values extracted from the signal collected with sensor A, green represents those extracted from sensor B, and blue illustrates values obtained from BH throughout all phases of the experimental protocol. In the lower panel, the mean values for all subjects, along with their standard deviations, are depicted across all phases of the experimental protocol. qb: quiet breathing; t: tachypnea; WS: wearable system (summed signal).

Below, the results of the $f_{R}$ values derived from the signals collected with the two developed flexible sensors (sensor A—depicted in orange, and sensor B—depicted in green) and the signal collected with the reference device (BH—depicted in blue) are shown. The results are presented in Figure 6 both individually for each subject and collectively as the mean across all subjects, including their respective standard deviations, throughout all phases of the experimental protocol.

Figure 6 shows that considering the mean value across all subjects, in the sitting position, there are lower errors for sensor A (max error 0.1 bpm) than for sensor B (max error 1.1 bpm). Similar behavior occurs when the subject is standing (max error sensor A: 0.3 bpm, max error sensor B: 3.2 bpm). Whereas during walking, the errors are lower for sensor B (error 0.1 bpm) than sensor A (1.9 bpm). The errors are comparable between the two sensors during running and stairs.

**Figure 7:**
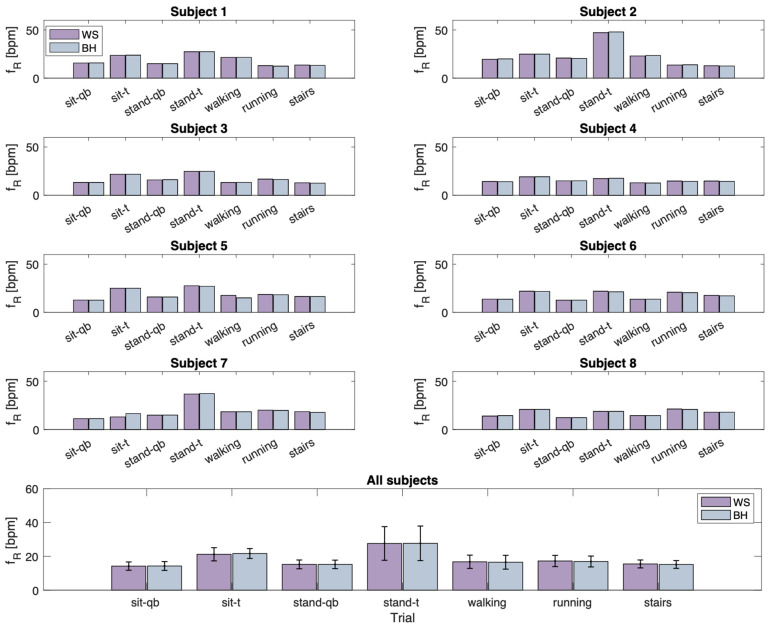
In the upper panels, bar charts are displayed for each subject. The color representation is as follows: purple corresponds to the $f_{R}$ values extracted from the summed signal (referred to as WS) and blue illustrates values obtained from BH throughout all phases of the experimental protocol. In the lower panel, the mean values for all subjects, along with their standard deviations, are depicted across all phases of the experimental protocol. qb: quiet breathing; t: tachypnea; WS: wearable system (summed signal).

Moreover, $f_{R}$ values were computed using a signal derived from the combination of signals collected with sensor A and sensor B (hereinafter, wearable system—WS). Therefore, Figure 7 illustrates the $f_{R}$ values extracted from the summed signal (referred to as WS and presented in purple) and the reference signal (referred to as BH and depicted in blue). This calculation was performed both individually for each subject and by combining the mean and standard deviations across all subjects.

It can be seen from Figure 7 that the errors are lower when $f_{R}$ values are calculated using the WS than for sensor A and sensor B. In fact, in this case, considering the average over all subjects, a maximum error of 0.5 bpm is obtained (subject sitting during tachypnea), which is much lower than the maximum error obtained with the previous analysis (3.2 bpm obtained for subject standing during tachypnea). During activities with more vigorous movement, the errors are comparable considering sensor A, sensor B, and the sum of the two sensors (errors between 0.3 bpm and 0.5 bpm).With regard to MAE, maximum values are obtained for sensor B during standing in tachypnea (3.2 bpm), higher than those obtained considering sensor A (max. 2.2 bpm) and the combination of the two sensors (max. 0.5 bpm).

## 5. Discussion and Conclusions

In this study, we carried out an analysis with a threefold objective. Initially, we examined the deformations observed in the chest wall during breathing, using an optoelectronic system equipped with passive photo-reflective markers and 10 infrared cameras. With this aim, 89 markers were placed on the rib cage according to a well-consolidated protocol. Next, the amplitude of the deformations between each pair of markers over time and the corresponding signal quality were analyzed and the two best positions were selected. This analysis enabled the identification of optimal measurement sites to place two piezoresistive textile sensors for $f_{R}$ monitoring. In addition, it provided insight into the dimensional requirements for the design and development of the sensors and provided guidance on their metrological characterization.

Subsequently, based on the results of the initial investigation, the piezoresistive sensors were designed, developed, and then embedded into a wearable t-shirt at defined rib cage locations using a polymer matrix to improve their robustness and flexibility and to decrease the influence of environmental factors on the sensing element. Subsequently, the sensors embedded into the wearable system were subjected to metrological characterization under both quasi-static and cyclic loads. The static characterization results showed a difference in static response between sensor A and sensor B at the same strain rate. This behavior could be due to differences in physical shape affecting the contact of the conductive fibers during sensor deformation, thus influencing the change in resistance. In addition, the parallel characterization of our sensors with the t-shirt fabric could contribute to these differences. However, this is a factor that is often overlooked in the literature, where sensors are typically characterized prior to integration, neglecting the deformation effect of the parallel fabric inherent in wearable respiratory monitoring devices. GF values of 6.0 and 5.8 for sensor A and sensor B, respectively, suggested a good sensor response to quasi-static loads, despite the integration of the piezoresistive textile into the t-shirt through the polymer matrix. These GF values are higher in comparison to other sensors suggested in the literature, such as capacitive strain sensors [29] (GF below 1.5 with 100% strain), weft knitted strain sensors [30] (GF of about 1 with 20% strain), and strain sensing threads [31] (GF of about 2.5 with 50% strain). Moreover, the obtained ${\%e}_{H}$ values, consistently below 25.3% in all cases, from the dynamic response of the flexible sensor to the application of repetitive ε at velocities simulating $f_{R}$ values (i.e., 6 bpm, 12 bpm, 24 bpm, and 36 bpm) affirmed the suitability of the proposed sensors for monitoring several $f_{R}$ ranges. In the literature, a few studies have proposed piezoresistive sensors encapsulated in flexible matrices made of Ecoflex [32] and two-component silicone rubber GMS 2628 [33] to develop wearable systems, but typically the placement of the sensors is performed without a preliminary study to optimize the $f_{R}$ measurement. The proposed systems were intended to be integrated into elastic bands using anchoring systems such as buttons.

Finally, a pilot study was carried out to assess the feasibility of the proposed system for monitoring $f_{R}$. Hence, eight healthy volunteers were enrolled, asked to perform both static (e.g., sitting, standing) and dynamic phases (e.g., walking, running, and climbing stairs), and simulate different breathing patterns (e.g., quiet breathing and tachypnoea). The system showed good agreement with the instrument used as a reference, as shown in Figure 6. In addition, the agreement with the reference device was also assessed by considering the sum of the signals taken from the two sensors in the wearable t-shirt. In this case, lower errors were found, underlining the advantage of having two different points on the rib cage for $f_{R}$ monitoring with average MAE values across all subjects and tests of 0.32 bpm, which were lower than MAE values obtained using sensor A (0.53 bpm) or sensor B (0.78 bpm).

The application of wearable systems, even under non-static conditions, poses challenges due to the presence of movements unrelated to breathing, which could induce artefacts [7]. The integration of a system consisting of two sensors proved useful as the proposed configuration allowed $f_{R}$ to be estimated even in situations where one of the two sensors did not adhere properly to the chest due to movements not related to breathing.

The strength of our study lies in the optimized positioning of the sensors on the chest wall, considering both the amplitude and the reliability of the collected signals. In addition, the integration of the sensors on the wearable solution by means of a polymer matrix not only allows for good adhesion between the sensor and the t-shirt, but also improves the robustness of the piezoresistive textiles and limits the influence of external factors that would lead to premature degradation of the mechanical and electrical properties of the sensors. Also, the metrological characterization introduces a novelty. This was undertaken after the sensors’ integration into the wearable system to enhance the reliability of assessing the calibration curve and dynamic behavior of the sensors. In fact, many studies merely characterize the sensor before integration, neglecting potential changes in metrological characteristics. Finally, the performance of the overall system is shown to be promising, even under non-static conditions. However, there are still some limitations to overcome. For example, a potential limitation stems from the variability in the participants’ builds, which may affect the accurate positioning of the sensors on the t-shirt. Although our t-shirt adjustment system mitigated this problem, sensor positioning errors due to anthropometric differences are still possible. The development of a signal quality index (SQI) could help to evaluate and improve the quality of the respiratory signal by addressing this limitation. Moreover, although in [34] the authors suggest that there are no significant differences in chest deformations between males and females during quiet breathing, our preliminary study of sensor placement choice was limited to male participants and further investigation is needed. Additionally, future developments could exploit the ability of the presented t-shirt to recognize activity based on changes in frequency over time, potentially extending its usability to recognize some vigorous activities while monitoring $f_{R}$ without other additional sensors. Moreover, the system could be enhanced with an inertial sensor for detecting the wearer’s movement, which, when supported by an activity recognition algorithm, could be useful in determining the reliability of respiratory data based on the wearer’s activity level. Lastly, for future research, the inclusion of finite element analysis (FEA) could be explored to enhance the understanding and optimization of sensor designs.

Looking forward, the developed piezoresistive textile sensors hold promise for several applications. Firstly, these sensors could be used for continuous respiratory monitoring in clinical settings, providing a non-intrusive means of tracking $f_{R}$ in patients with respiratory disorders or undergoing respiratory rehabilitation. They can also facilitate early detection of respiratory abnormalities and support remote patient monitoring. In addition, the integration of additional physiological sensors, such as heart rate monitoring systems or accelerometers, could lead to more comprehensive health and fitness monitoring.

## Figures and Tables

**Table 1:** Results for $S_{\varepsilon \;}$(5%) and $S_{\varepsilon }\;$(10%) obtained applying a quasi-static tensile load to the two developed flexible sensors (displacement rate: 5 mm/min). *: mean value.

Sensor	$S_{\varepsilon }$ (5%)	Load * (5%)	*GF* (5%)	$S_{\varepsilon }$ (10%)	Load * (10%)	*GF* (10%)
Sensor A	9.4 $\Omega \cdot {\%}^{-1}$	1.4 N	6.0	7.2 $\Omega \cdot {\%}^{-1}$	2.6 N	4.7
Sensor B	9.1 $\Omega \cdot {\%}^{-1}$	1.7 N	5.8	5.3 $\Omega \cdot {\%}^{-1}$	3.1 N	3.2

**Table 2:** Results for ${\%e}_{H}$ obtained applying 30 loading and unloading cycles to the two developed flexible sensors.

Sensor	6 bpm	12 bpm	24 bpm	36 bpm
Sensor A	24.9%	23.0%	20.8%	20.4%
Sensor B	25.3%	25.1%	21.4%	21.2%

## Data Availability

The data presented in this study are available upon request from the corresponding author. The data are not publicly available due to privacy reasons.
